# Quantitative Magnetic Resonance Imaging (qMRI) of the Small Bowel in Crohn's Disease: State‐of‐the‐Art and Future Directions

**DOI:** 10.1002/jmri.29511

**Published:** 2024-07-06

**Authors:** Naomi S. Sakai, Timothy J.P. Bray, Stuart A. Taylor

**Affiliations:** ^1^ Centre for Medical Imaging University College London London UK

**Keywords:** Crohn's disease, inflammation, fibrosis, quantitative MRI

## Abstract

Crohn's disease (CD) is a chronic inflammatory disease of the gastrointestinal tract in which repeated episodes of acute inflammation may lead to long‐term bowel damage. Cross‐sectional imaging is used in conjunction with endoscopy to diagnose and monitor disease and detect complications. Magnetic resonance imaging (MRI) has demonstrable utility in evaluating inflammatory activity. However, subjective interpretation of conventional MR sequences is limited in its ability to fully phenotype the underlying histopathological processes in chronic disease. In particular, conventional MRI can be confounded by the presence of mural fibrosis and muscle hypertrophy, which can mask or sometimes mimic inflammation. Quantitative MRI (qMRI) methods provide a means to better differentiate mural inflammation from fibrosis and improve quantification of these processes. qMRI may also provide more objective measures of disease activity and enable better tailoring of treatment. Here, we review quantitative MRI methods for imaging the small bowel in CD and consider the path to their clinical translation.

**Level of Evidence:**

2

**Technical Efficacy:**

Stage 2

Crohn's disease (CD) is a chronic inflammatory bowel disease that can affect any part of the gastrointestinal tract, but particularly affects the terminal ileum and approximately one third of patients present with disease limited to the ileum.^1^ It is a relatively frequent condition with highest prevalence in Europe (322 per 100,000 people in Germany) and North America (319 per 100,000 in Canada).^2^ Incidence has been rising over the last 30 years in newly industrialized countries in Africa, Asia, and South America.^2^ CD particularly affects adolescents and young adults with most cases diagnosed in those aged 15–25 years.^3^ There are a number of different disease phenotypes but, in some, the disease course is progressive with recurrent bowel inflammation and tissue remodeling over time leading to mural collagen deposition, intestinal fibrosis, and muscle hypertrophy, causing luminal narrowing and potentially leading to obstruction, which may ultimately require surgical intervention.^4^ Since inflammation precedes and drives fibrosis, the management of CD has evolved to a treat‐to‐target paradigm where objective treatment targets are defined with the aim of improving outcomes and reducing end‐organ damage such as development of a stricture or fistula. Once targets are defined (eg, biochemical markers, endoscopic findings), patients have frequent objective assessments to monitor disease activity and optimize therapy to reach the therapeutic goal.

A key aspect of monitoring in CD is distinguishing between active inflammation and chronic tissue damage and fibrosis. Inflammatory lesions are managed with anti‐inflammatory medical therapy, while fibrosis is managed with endoscopic dilation or surgical resection. Although symptoms, endoscopic findings, serum markers and stool inflammatory markers, or clinical scoring system such as Crohn's disease activity index (CDAI) are used to assess the disease activity, cross‐sectional imaging including magnetic resonance enterography (MRE) plays a central role in the diagnosis and monitoring of disease activity in CD.

MRE is preferred to CT enterography or endoscopy as it enables noninvasive evaluation of the entire digestive tract without ionizing radiation, is well tolerated by patients, and is now readily available.^5^ It also enables identification of extraluminal complications and can visualize segments upstream of terminal ileum.

The aims of MRE in CD are to detect disease and complications such as penetrating disease; to identify and distinguish active inflammation and fibrosis; and to monitor treatment response. Signal abnormalities on MRI are linked to inflammatory change or fibrosis.^6^ However, a major challenge for imaging in CD is differentiating inflammation and fibrosis as the pathologies usually coexist and MRE findings may overlap.^7^ A solution to this may be to use quantitative MRI (qMRI) techniques which can separate the different pathological processes and at the same time remove the subjectivity of image interpretation. In this review, we summarize different qMRI techniques that have been investigated in the assessment of disease in CD and discuss the added value of qMRI to overcome the limitations of conventional MRE.

## Conventional MRE

### Conventional MRE Protocol for CD

**Figure 1:**
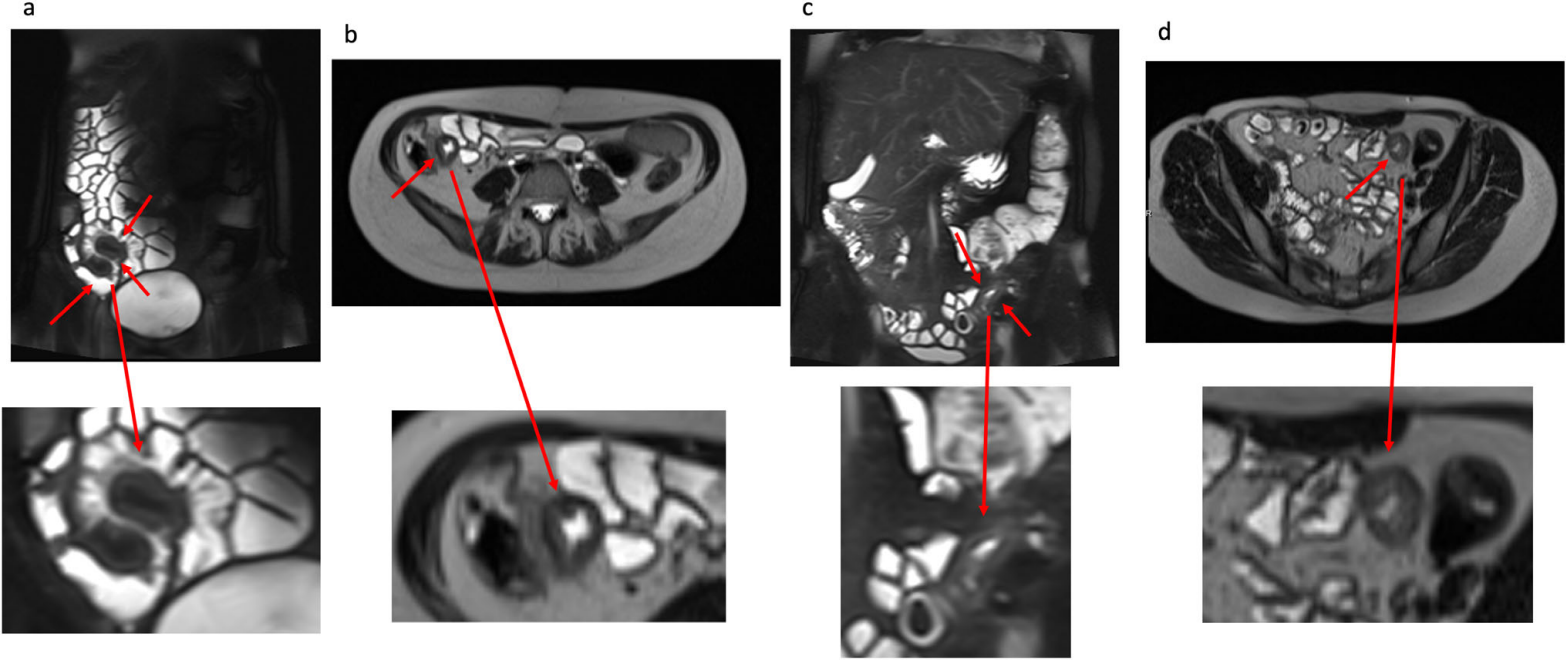
Example conventional images demonstrating inflammation in two patients with active CD. (**a**) Coronal fat‐suppressed SSTSE and (**b**) axial SSTSE images with marked bowel wall thickening and mural edema with increased T2 signal in the bowel wall indicating inflammation of the terminal ileum (red arrows); (**c**) coronal fat‐suppressed SSTSE and (**d**) axial SSTSE images in a different patient also demonstrating mural thickening and edema (increased T2 signal) of the distal ileum in consistent with inflammation (red arrows). Corresponding magnified images of the abnormality are displayed underneath each image. SSTSE = single‐shot turbo spin‐echo.

Currently, MRE relies on three sequences: 1) a balanced steady‐state free precession (SSFP) (eg, True FISP, FIESTA) for assessment of mesenteric changes such as hypervascularity and fibrofatty proliferation; 2) T2‐weighted fast spin echo imaging with and without fat suppression for detecting bowel wall edema (Fig. 1); and 3) three‐dimensional T1‐weighted volumetric gradient echo imaging pre‐ and postcontrast with fat suppression to evaluate the level and pattern of bowel wall enhancement. As practice has evolved, many institutions now also routinely include diffusion‐weighted imaging (DWI), which is performed using echo planar readouts (with at least one low *b*‐value [*b* = 0–50 seconds/mm^2^] and one high *b*‐value [*b* = 800–1000 seconds/mm^2^] acquisition), and some no longer routinely administer gadolinium (including our own institution where motility sequences are included instead). Patients are given an antispasmodic (eg, glucagon, hyoscine butylbromide [Buscopan]) to reduce bowel wall motion and water‐based contrast agents (eg, mannitol, polyethylene glycol [MiraLAX], barium sulfate [VoLumen]) are used to distend the small bowel lumen to better depict mural abnormalities.

### Limitations of Conventional MRE for Assessing CD

MRE enables assessment of the disease burden in terms of anatomical distribution, transmural extent, and complications, and also allows visualization of extra‐intestinal features including mesenteric inflammatory changes. Mural thickness, T2 hyperintensity, and contrast enhancement patterns show the disease extent on conventional MRE. However, conventional MRI sequences used in MRE are limited by the fact that the assessed MRE findings are not specific to the disease process such as inflammation or fibrosis. Differentiating between inflammation and fibrosis is particularly challenging because the two often coexist in patients with CD.^7^ This distinction has important therapeutic implications.

Fibrosis may be assumed to be present when there is bowel wall thickening but absence of imaging features of inflammation such as T2 hyperintensity or avid mural enhancement. However, in a patient with both active and chronic inflammation, the bowel thickening observed in fibrosis could be misinterpreted as part of the active inflammation leading to overestimation of the acute component. Conversely, imaging features of acute inflammation such as mural edema and enhancement may be diminished by the presence of fibrosis. The potential underestimation of activity could result in missing opportunities to use appropriate medical therapy promptly to prevent irreversible damage.

This problem is compounded by the fact that MRE interpretation is subjective and dependent on expertise. Despite consensus guidelines on reporting of imaging in CD,^8^, ^9^ there are inconsistencies between readers, particularly regarding the extent of disease showing no to fair agreement between the readers.^10^

### Improving the Interpretation of MRE Using Semiquantitative MRE Activity Scores

MRE activity scores are semiquantitative and aim to make the assessment of MRE findings more objective and systematic.^11^, ^12^

The first and best‐validated MRE activity score is the Magnetic Resonance Index of Activity (MaRIA) score which was derived using CD Endoscopic Index of Severity (CDEIS) as a reference standard. It is calculated from four observations: wall thickness, quantified mural contrast enhancement, mucosal ulceration, and mural T2 signal intensity (edema).^13^, ^14^ The Clermont score is derived from the MaRIA score but includes the apparent diffusion coefficient (ADC) instead of contrast enhancement,^15^ thus removing the need for intravenous contrast administration.

The simplified MaRIA (sMaRIA)^16^ is based on the MaRIA score and uses four observations: wall thickness, T2 signal intensity (edema), fat stranding, and mucosal ulceration. Notably it does not require contrast‐enhanced imaging and requires less time to calculate than either MaRIA or Clermont scores and therefore has more potential utility in clinical practice.^17^ A further scoring system, the London score, is relatively simple to measure, grading mural thickness and mural T2 signal intensity.^18^ The Magnetic Resonance Enterography Global Score further extends this with the aim of assessing the true extent of disease by including disease length and complications such as fistulae and abscesses and has shown good correlation with fecal calprotectin levels.^19^

MRE activity scores have been shown to have good interobserver agreement in several studies with comparable agreement between the different scoring systems showing intraclass correlation coefficients (ICC) of between 0.70 and 0.74.^20^ Scoring systems also demonstrated similar correlation to CDEIS.^20^ Both the sMaRIA and London scores have been shown to achieve high sensitivity for active disease in the terminal ileum when compared with terminal ileal biopsy results: sensitivity of 83% (95% confidence interval [CI]: 74%–90%) for sMaRIA and 76% (95% CI: 67%–84%) for the London score. However, their specificity against histology is low (sMaRIA 41% [95% CI: 23%–61%]; London 64% [95% CI: 43%– 80%]),^12^ which may be partly due to the limitations of the superficially biopsied reference standard compared to the transmural assessment achieved with MRE.

Despite their promise, MRE scoring systems are not widely used in clinical practice and their use is largely reserved for research. The scoring systems remain limited by the fact that MRE itself is imperfect with imaging findings not being specific to the disease process, i.e., inflammation or fibrosis and interpretation being subjective.

## Quantitative MRI for CD Assessment

While the use of MRI‐based CD scoring systems removes some of the subjectivity of image assessment by ensuring a systematic approach and enables comparison of disease activity at different timepoints, qMRI techniques offer a potentially more objective and less variable approach and could enable differentiation between inflammation and fibrosis.

With conventional MRI, the signal derived from the bowel and extra enteric tissues not only depends on the target process such as inflammation, but also on other tissue factors including T1 value, proton density, diffusivity, and other nontissue factors such as MRI hardware and acquisition parameters. To mitigate this, qMRI aims to measure tissue properties more directly and can potentially disentangle the different tissue effects so that the target process is isolated. At the same time the influence of MRI hardware and acquisition parameters are minimized.

The qMRI requires three key ingredients: 1) a succession of images acquired with different scanner settings; 2) a mathematical model describing the relationship between tissue properties (qMRI parameters), scanner settings, and the signal expected in a voxel; and 3) an algorithm that finds the parameter values producing the minimum error (or maximum likelihood) between the predicted signals (based on the parameter estimates) and the measured signals. Having derived estimates of the parameters in each voxel, these estimates are combined to generate a parameter map. The quantitative information is typically extracted from the parameter maps using a region of interest (ROI) which is placed onto an abnormal (or normal) area. Summary metrics can be derived from the numerical value of each voxel in the ROI using different methods, the simplest being an average of all voxels in the ROI. The summary metrics from the parameter maps can be taken as quantitative imaging biomarkers (QIBs).^21^ In CD, QIBs have the potential to separate inflammation and fibrosis. qMRI can also eliminate the subjectivity of image interpretation because it uses numerical values from parameter maps rather than a visual assessment of image contrast.

## Quantitative MRI Techniques in Imaging of CD

Various qMRI techniques have been explored in CD, several of which enable assessment of disease activity and treatment monitoring without the use of intravenous contrast. The QIBs derived from each of the qMRI techniques are designed to target different pathophysiological process; this is summarized in Table 1. A summary of studies of qMRI in CD with diagnostic performance against histopathological, imaging, or clinical endpoints is shown in Table 2.

**Table 1:** Summary of qMRI Techniques With Potential Utility in Imaging of Small Bowel Crohn's Disease

qMRI Technique	Biomarker	Target Pathophysiological Process	Pros	Cons
Diffusion weighted imaging	ADC	Inflammation Fibrosis	High availability	Low spatial resolution; low SNR; sensitive to motion
	IVIM (pure diffusion D, perfusion fraction *f*, pseudodiffusion coefficient D*)	Inflammation (D) Fibrosis (*f*)	Non‐invasive measure of dynamic properties	Less availability; low SNR; sensitive to motion
Relaxometry	T1	Fibrosis	High availability; high spatial resolution	Susceptible to confounding factors
	T2	Inflammation		
	T2*	Fibrosis		
Dynamic contrast‐enhanced imaging (DCE)	Pharmacokinetic perfusion parameters, eg, signal intensity curve, RE, ME, TTP, and BE	Inflammation Fibrosis	High availability; dynamic measurements	Intravenous contrast
Magnetization transfer imaging	MTR	Fibrosis	Improved contrast	Lack of specificity; less availability; low SNR; high SAR
Motility	Motility index	Bowel motility	Measures bowel function	Requires specialist postprocessing
Elastography	Stiffness	Fibrosis	Targets fibrosis; used in other organs with success	Lower spatial resolution; susceptible to noise; requires specialist equipment

ADC = apparent diffusion coefficient; BE = brevity of enhancement; DCE‐MRI = dynamic contrast enhanced MRI; DWI = diffusion‐weighted imaging; IVIM = intravoxel incoherent motion; ME = maximum enhancement; MTR = magnetization transfer ratio; RE = relative enhancement; SAR = specific absorption ratio; SNR = signal‐to‐noise ratio; TTP = time to peak.

**Table 2:** Summary of Studies of qMRI in Crohn's Disease With Diagnostic Performance Against Reference Standard

Study	*N*	Technique	Endpoint	Reference Standard	AUROC
Oto et al^22^	18	DCE‐MRI DWI	Inflammation	Histopathology (endoscopy within 2 months of MRE)^a^	*K* ^trans^: 0.92 *v*_e_: 0.88 ADC: 0.92 ADC + *K* ^trans^: 0.95
Rimola et al^23^	41	DCE‐MRI	Fibrosis	Histopathology^b^	Enhancement between 70 second and 7 minutes: 0.93
Lee et al^24^	30	DCE‐MRI	Inflammation	Histopathology on biopsy from endoscopy^c^	*K* ^trans^: 0.864 *K* ^ep^: 0.619 *v*_e_: 0.685
Coimbra et al^25^	60	DCE‐MRI DWI (ADC) Magnetization transfer imaging	Fibrosis Inflammation	Histopathology (surgical specimens)^d^	Moderate vs. severe fibrosis ADC: 0.894
Hectors et al^26^	27	DCE‐MRI IVIM‐DWI	Inflammation	Abnormal vs. normal bowel segments defined on MRE	*K* ^trans^ + *v*_e_ + PF + ADC: 0.963 ADC: 0.8
Li et al^27^	30	DWI	Fibrosis	Histopathology (surgical specimens)^e^	No/mild fibrosis vs. moderate/severe fibrosis in mildly inflamed bowel ADC: 0.867
Caruso et al^28^	30	DWI	Fibrosis	Histopathology (surgical specimen)^f^	ADC: 0.83
Hectors et al^26^	27	DCE‐MRI DWI	Inflammation	Abnormal vs. normal bowel segments based on inflammation on MRE	*C*_peak_: 0.733 AUC: 0.733 ADC: 0.800; *K* ^trans^ + *v*_e_ + PF + ADC: 0.963
Buisson et al^15^	31	DWI	Inflammation	MaRIA score	ADC: 0.96
Buisson et al^29^	40	DWI	Response to anti‐TNF (change in inflammation)	CDAI, CRP	Remission at 12 weeks ADC: 0.703
Li et al^30^	43	DWI	Inflammation	Endoscopy SES‐CD	ADC: 0.973
Wagner et al^31^	35	DWI	Inflammation Fibrosis	Histopathology (surgical specimen)^g^	Inflammation ADC: 0.728 Fibrosis vs muscular hypertrophy ADC: 0.556
Li et al^32^	31	Magnetization transfer imaging DWI	Fibrosis	Histopathology (surgical specimen)^e^	Presence of fibrosis: MTR: 0.981 ADC: 0.869 No/mild vs moderate/severe fibrosis: MTR: 0.919 ADC: 0.747
Menys et al^33^	82	Motility	Inflammation	Endoscopic CDEIS Histopathologic EAIS	TI motility: 0.86 (against CDEIS) TI motility: 0.87 (against EAIS)
Zhang et al^34^	24	IVIM‐DWI	Fibrosis	Histopathology (surgical specimen)^h^	No/mild vs. moderate/severe fibrosis: PF: 0.876 ADC: 0.802
Huang et al^35^	27	Relaxometry (T2*)	Fibrosis	Histopathology (surgical specimen)^i^	No/mild fibrosis vs. moderate/severe fibrosis: T2*: 0.951
Meng et al^36^	20	Magnetization transfer imaging	Fibrosis	Histopathology (surgical specimen)^i^	Mild/moderate vs. severe fibrosis: MTR: 0.895
Fang et al^37^	28	Magnetization transfer imaging	Fibrosis	Histopathology (surgical specimen)^e^	MTR: Mild vs. moderate/severe fibrosis: MTR: 0.964
Pazahr et al^38^	31	Magnetization transfer imaging	Fibrosis	MRI clinical activity score	No vs. presence of fibrosis MTR: 0.98
Avila et al^39^	69	Elastography	Fibrosis	Visual analog scale^j^	Occurrence of clinical event 0.82^k^
Reiter et al^40^	40	Elastography	Fibrosis	Histopathology (surgical specimen)^l^	Prediction of IBD SWS: 0.90 Loss angle: 0.84

ADC = apparent diffusion coefficient; AUROC = area under receiver operating characteristic; CDEIS = Crohn's disease endoscopic index of severity; C_peak_ = peak concentration; DCE‐MRI = dynamic contrast enhanced MRI; DWI = diffusion‐weighted imaging; EAIS = endoscopic biopsy acute histologic inflammatory score; IVIM = intravoxel incoherent motion; *K* ^ep^ = wash‐out constant; *K* ^trans^ = volume transfer constant between the intravascular space and extravascular space; MRE = MR enterography; MTR = magnetization transfer ratio; PF = perfusion fraction; SES‐CD = simple endoscopic score; SWS = shear wave speed; TI = terminal ileum; TNF = tumor necrosis factor; *v*_e_ = volume of extravascular space per unit volume of tissue.

^a^ Patients with endoscopic of histopathologic findings of active disease. Endoscopic findings indicative of active disease were erosions, ulceration, granularity, or friability. Histopathologic findings of active disease were the presence of crypt abscesses, mucosal ulceration, neutrophilic infiltration, and edema.

^b^ Pathologic microscopic data included the fibrosis (Chiorean Score) and inflammation scores (three‐level score), the presence of stenosis, fistulae, and ulcers, and wall thickness.

^c^ Active Crohn's disease defined as the presence of neutrophil infiltrations in the cryptal/surface epithelium or lamina propria of the biopsy specimen. Inactive Crohn's disease defined as the presence of crypt architectural distortion or crypt atrophy at the biopsy specimen without neutrophil infiltration. The severity of inflammatory lesions was graded on a scale from 0 to 3.

^d^ Inflammation and fibrosis were evaluated using a Modified Chiorean Score.

^e^ Inflammation and fibrosis were graded on a scale from 0 to 3.

^f^ Acute Inflammatory Score for inflammation and Fibrosis Score for fibrosis.

^g^ Active inflammation was graded on a scale from 0 to 3, based on the depth of neutrophil infiltrates. Fibrosis was measured based on collagen deposition.

^h^ Bowel fibrosis was graded as none, mild, moderate, or severe.

^i^ Fibrosis was graded on a scale from 0 to 3.

^j^ Visual analog scale (VAS) from 0 to 9 corresponding to the degree of fibrosis estimated by the radiologist; score 0 = no fibrosis.

^k^ Clinical event defined as: abdominal surgery, hospitalization, or consultation at emergency department for abdominal pain or digestive occlusion within 450 days of MRI.

^l^ Diagnosis of Crohn's disease or ulcerative colitis confirmed using histopathological analysis of surgical specimens.

### Diffusion‐Weighted Imaging

Diffusion‐weighted imaging (DWI) exploits differences in the freedom of water diffusion in tissues, known as diffusivity, to generate signal contrast. In DWI, a series of images is acquired with different degrees of diffusion weighting, introduced using motion‐probing gradients, which are typically added to a spin echo sequence. The degree of diffusion weighting is summarized using the *b*‐value. Typically, a range of *b*‐values is acquired, including *b* = 0 (no diffusion weighting) through to higher *b*‐values (increased diffusion weighting). On the higher *b*‐value images, the signal in areas of free diffusion is attenuated, while in regions where diffusion is restricted the signal is less attenuated. If $S_{0}$ is the MR with *b* = 0 and *D* is the diffusion coefficient, the signal *S* after the diffusion gradients have been applied is given by:

$$S=S_{0}e^{-\mathrm{bD}}$$

Here the diffusion coefficient *D* provides a measure of tissue diffusivity and is frequently referred to as the ADC. The use of at least two *b*‐values is required for the ADC calculation, typically including a lower value of 0–50 seconds/mm^2^ and a higher value of 800–1000 seconds/mm^2^. Importantly, whereas the diffusion‐weighted images retain contrast that depends on other tissue properties, the ADC maps can provide a more targeted assessment of the diffusivity of the tissue, independent of these other confounders.

**Figure 2:**
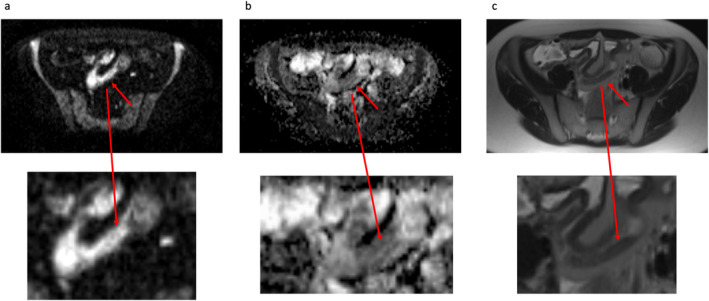
DWI in active CD in the terminal ileum. (**a**) axial DWI image (*b*‐value = 600 seconds/mm^2^) with increased signal intensity in in the abnormal ileum (red arrow); (**b**) the ADC_0‐600_ map demonstrates corresponding low ADC in the abnormal ileum in keeping with restriction of diffusion; (**c**) conventional T2 HASTE image for reference demonstrating the thickened, inflamed ileum. Corresponding magnified images of the abnormality are displayed underneath each image. DWI = diffusion‐weighted imaging; CD = Crohn's disease; ADC = apparent diffusion coefficient; HASTE = half‐Fourier acquisition single‐shot turbo spin‐echo.

ADC values have been shown to be reduced in inflamed segments of bowel in CD in a number of studies, many using histopathological samples as the reference standard^41^, ^42^ (example in Fig. 2). In active CD, the wall of the small bowel is infiltrated by inflammatory cells which, together with dilation of lymphatic channels, is thought to cause restriction of diffusion of water molecules.^43^ Several studies have, however, also demonstrated that ADC values are also reduced in fibrotic tissue, thought to result from a reduction in the extracellular space caused by excessive extracellular matrix collagen deposition.^28^, ^42^ Caruso et al^28^ found that ADC was negatively correlated with the fibrosis score (*r* = −0.648; *P* < 0.0001) and could be useful to identify fibrosis in CD. However, since both inflammation and fibrosis have been shown to reduce ADC values, DWI may be less useful where the two coexist. Li et al^27^ found significant inverse correlation between ADC and bowel fibrosis in mildly inflamed segments (*r* = − 0.641, *P* = 0.001) but not in areas of moderate or severe inflammation, suggesting that ADC is able to quantify fibrosis only where there is little inflammation present.

Intravoxel incoherent motion (IVIM) is an alternative approach to diffusion MRI which models the tissue as a “tissue” compartment and a microvascular, “pseudo‐diffusion” compartment, thus allowing the extraction of perfusion or perfusion‐like information.^44^ While diffusion refers to the random movement of individual molecules which results in signal attenuation in the presence of a magnetic field, pseudo‐diffusion refers to blood flow resulting from collective water flow in randomly oriented capillaries. The vascular, pseudo‐diffusion component decays much faster, enabling diffusion and pseudodiffusion to be separated with the overall signal attenuation being the sum of the tissue (diffusion) and blood (pseudodiffusion) components. The IVIM model can be expressed as:

$$S=S_{0}\left(f_{\text{IVIM}}e^{-b\left(D_{\text{tissue}}+D^{*}\right)}+\left(1-f_{\text{IVIM}}\right)e^{-bD_{\text{tissue}}}\right)$$

where *f* is the perfusion fraction (the percentage of a voxel occupied by capillaries); 1 − *f* therefore reflects the fractional contribution of the tissue compartment; D* is the pseudodiffusion coefficient; and $D_{\text{tissue}}$ is the tissue diffusion coefficient. Note that the exponent in the first term is $D_{\text{tissue}}+D^{*}$ rather than simply $D^{*}$; this effectively constrains the total diffusivity for the pseudodiffusion compartment to be greater than that in the tissue compartment, and thus ensures that the diffusivity values for the two compartments are correctly assigned.

As pseudodiffusion is observed at low *b*‐values, the acquisition of at least three *b*‐values is necessary for IVIM‐DWI, although typically a larger number (8–16) of *b*‐values were acquired in the literature. Since acute inflammation and fibrosis both result in changes in perfusion (increased and decreased, respectively), IVIM‐DWI represents an attractive method to distinguish the processes as it enables an estimation of perfusion/blood flow without the need for intravenous contrast. Zhang et al^34^ found that perfusion fraction was negatively correlated with fibrosis scores and outperformed ADC in grading fibrosis. However, two studies have also demonstrated a reduction in the perfusion fraction in inflamed bowel.^26^, ^45^ This seems counterintuitive but could be explained by the fact that perfusion fraction represents the proportion of a voxel occupied by capillaries (i.e., measures vascularity not flow); this is supported by a study of surgical specimens which demonstrated reduction of microvascular volume in affected bowel segments in patients with CD.^46^ Alternatively, this could relate to increase tissue diffusivity (making D* and *D*_tissue_ more similar) or to an increase in extracellular water (reducing the fractional contribution of the pseudodiffusion component). Previous work by our group in bone marrow found a reduction in the perfusion fraction $f_{\text{IVIM}}$ in inflamed bone marrow (“bone marrow edema”) compared to normal bone marrow (i.e., the signal became closer to monoexponential),^47^ suggesting that there could be similar biophysical changes occurring in both tissues.

A potential alternative to the perfusion fraction may be the blood flow‐related parameter *f*_IVIM_ D* (i.e., the product of the perfusion fraction and the pseudodiffusion coefficient, which itself holds information on blood speed), which gives information on the quantity of blood flowing through a unit tissue per unit time. However, it is also worth noting that in general, the relationship between perfusion and D* remains unclear; in addition to the findings discussed earlier in the bowel wall and bone marrow, studies using IVIM to assess liver fibrosis have also demonstrated varying changes in perfusion fraction, D* and *D*_tissue_.^48^ The potential utility of IVIM therefore remains uncertain until we have a greater understanding of how the measured components relate to physiology.

Although valuable and widely used in clinical practice, the quantitative approach of DWI has limitations due to its low spatial resolution, motion artifacts, and often observed low signal‐to‐noise ratio. ROI measurement in a small target segment is often subject to inter‐ and intraobserver variabilities and the variability of perfusional parameters (D* and *f*) of IVIM‐DWI have been reported.^26^, ^34^, ^45^ Fasting prior to scanning, the use of antispasmodics, and the use of simultaneous multislice or multiband DWI may mitigate motion artifacts.

### MR Relaxometry (T1, T2, and T2* Relaxation)

MR relaxometry refers to measuring relaxation times, such as T1, T2, or T2*, from a series of MR images. T1, T2, and T2* imaging, respectively, have shown relationships with myocardial fibrosis, edema, and iron deposition in cardiac imaging.^49^ Since inflammation in CD is characterized by bowel wall edema, T2 and T2* have been investigated more frequently than T1 mapping. Given its uses in other organs, T1 mapping may be more useful for assessing fibrosis in the bowel.

**Figure 3:**
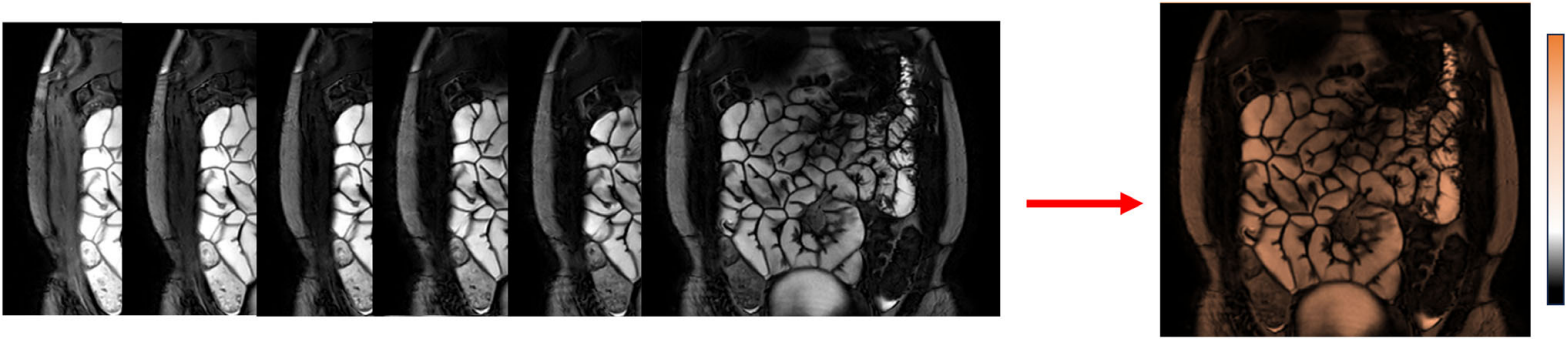
Schematic of generation of a T2 map of the abdomen. Coronal images of the abdomen with different echo times (TE) to measure the T2 decay are used to generate the T2 map. (Images courtesy of Dr Caroline Hoad, University of Nottingham.)

A Carr‐Purcell‐Meiboom‐Gill spin‐echo sequence which involves taking measurements at different echo times in an echo train to sample the T2 decay curve is the gold standard for T2 mapping (Fig. 3). However, sampling the whole T2 decay curve is relatively time consuming and this method is affected by signal contamination from stimulated echoes. A simpler implementation is to acquire multiple spin‐echo images with different echo times but the scan durations become prohibitively long for clinical use. T2 weighting can be introduced through several methods including varying the echo time and the addition of T2 preparation pulses (with varying T2 preparation times). With the T2 preparation pulses, the longitudinal magnetization is prepared with a “pre‐pulse” in advance of the excitation radiofrequency (RF) pulse.^50^, ^51^ The T2 preparation approach may have the advantage of being less susceptible to artefact such as motion because the duration of the readout component of the sequence is potentially reduced.

Since histopathological abnormalities in CD may involve both mural edema and fat deposition, consideration of the potential confounding effect of fat is important for relaxometry methods. This is important because fat has a different T2 relaxation time and can therefore bias the qMRI parameter estimates if not accounted for. Recently, “Dixon‐relaxometry” methods have been used to first separate water and fat and then derive water‐ and fat‐specific T2 measurements, as well as fat fraction measurements, offering a multiparametric quantitative assessment of multiple inflammatory processes.^52^ This may be applicable to bowel wall imaging where the presence of fat (both adjacent to the bowel wall and potentially within the wall itself following inflammation) is also important.

T2 mapping has been used in assessing the permeability of the bowel wall^53^; increased gut permeability has been implicated in the pathophysiology of CD where it is seen before macro‐ and microscopic manifestation of the disease^54^ and is reversible with biologic therapy.^55^ In a study of healthy volunteers, qMRI measures of small bowel T2 were significantly increased following provocation and correlated with measures of gut permeability.^53^ In a feasibility study, T2 mapping using the technique of MR fingerprinting demonstrated increased T2 relaxation times in segments of bowel with active inflammation identified on conventional imaging in patients with CD.^56^ T2 relaxation time has also been investigated in the assessment of response to therapy in perianal CD where the baseline T2 relaxation time showed moderate ability to predict response to biologic treatment.^57^

T2* relaxation refers to decay of transverse magnetization seen with gradient‐echo sequences caused by the combined effect of T2 relaxation and magnetic field inhomogeneity (T2′), i.e., $\frac{1}{T_{2}*}=\frac{1}{T_{2}}+\frac{1}{{T_{2}}^{\prime }}$, which includes susceptibility‐induced field distortions produced by the tissue being imaged. Susceptibility differences among tissues secondary to increasing paramagnetic material (eg, caused by the presence of iron, calcium, collagen) lead to faster T2* relaxation, leading to signal loss. T2* measurements are typically performed using a spoiled gradient echo sequence with multiple echoes.

The susceptibility sensitivity of T2* means that it offers a method to assess fibrosis by exploiting the increased collagen deposition seen in repeated bowel wall damage.^58^ Huang et al^35^ demonstrated that T2* decreased with increasing fibrosis in the bowel wall and found a moderate correlation between T2* values and histological fibrosis scores in 27 patients with CD. T2* mapping without contrast administration outperformed CE imaging in assessing and grading intestinal fibrosis with significant differences in the T2* values between mild, moderate, and severe fibrosis. A combination of T2 and T2* may therefore enable separation and measurement of bowel wall edema and fibrosis in CD. T2* measurements have shown excellent interobserver agreement (ICC 0.893).^35^

Many techniques for T1 mapping have been developed, the traditional gold standard method being a series of independent single‐point inversion recovery signal measurements at different TIs. However, this is too time consuming for clinical use and other techniques such as the Look‐Locker technique in which multiple signal measurements are acquired after an inversion pulse (generating multiple images along the T1 recovery curve each with a well‐defined TI) and variable flip angle have since been developed to overcome this. T1 mapping has been shown to correlate with the degree of fibrosis (assessed using a visual analog scale) in a study of 33 patients with CD.^59^

### Dynamic Contrast‐Enhanced Imaging

**Figure 4:**
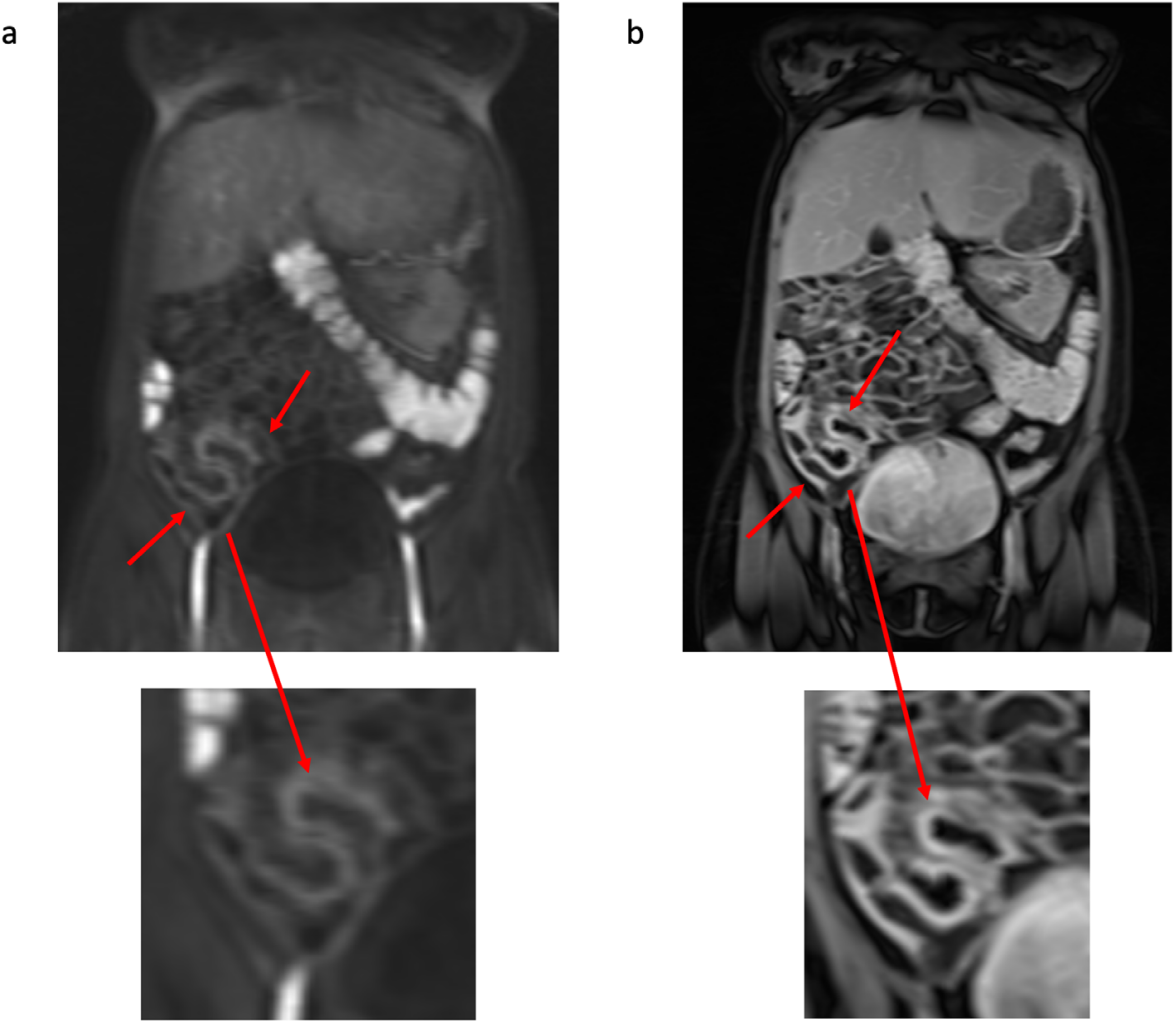
Example of DCE images from MRE in a patient with terminal ileitis due to CD. (**a**) An ileal segment with mucosal enhancement acquired 70 seconds after gadolinium injection (red arrows) and (**b**) progressive hyperenhancement on the delayed images 5 minutes after injection (red arrows). Corresponding magnified images of the abnormality are displayed underneath each image. DCE = dynamic contrast‐enhanced; MRE = magnetic resonance enterography; CD = Crohn's disease.

Dynamic contrast‐enhanced MRI (DCE‐MRI) measures T1 changes in tissues over time after administration of an intravenous gadolinium‐based contrast agent and modeling of the data allows quantification of tissue vascular properties. While relative enhancement is measured in standard contrast‐enhanced MRE, DCE‐MRI enables measurement of both quantitative pharmacokinetic model and model‐free semiquantitative parameters. DCE‐MRI protocols vary slightly but typically use gadolinium‐based contrast at a dose of 0.1–0.2 mmol/kg at a rate of 2 – 5 mL followed by a saline flush of approximately 20 mL.^22^, ^60^, ^61^ Multiple acquisitions are then performed in rapid succession enabling temporal changes in signal intensity to be measured (temporal resolution is typically 5–12 seconds) (Fig. 4).

A simple two‐compartment model of the intravascular extracellular space and extravascular extracellular space is used to describe the distribution of the gadolinium after the injection (the gadolinium may be present in either the intravascular space, i.e., true perfusion, or the extravascular space, i.e., increased permeability). The model predicts the change in contrast concentration *C*(*t*) as a function of time, and can be written as:

$$\frac{dC\left(t\right)}{dt}=K^{\text{trans}}\times \left[C_{p}\left(t\right)-\frac{C\left(t\right)}{v_{e}}\right]$$

where *K* ^trans^ (minute^−1^) is the volume transfer constant between the intravascular space and extravascular space, *v*_e_ is the volume of extravascular space per unit volume of tissue, and *C*_*p*_(*t*) is the arterial input function. *K* ^ep^, the wash‐out constant, is equal to the *K* ^trans^ divided by *v*_e_. Thus, pharmacokinetic modeling of DCE‐MRI data can separate mural perfusion (parameters *K* ^trans^ and *K* ^ep^) from vascularity (*v*_e_).^62^ Semiquantitative parameters including initial area under the curve (IAUC), initial slope of enhancement, time to peak enhancement (*T*_peak_), and peak concentration (*C*_peak_) can also be measured.

DCE‐MRI‐derived parameters have been investigated for evaluation of CD activity,^22^, ^24^, ^26^, ^41^, ^42^ fibrosis,^23^, ^25^, ^42^ and assessment of treatment response.^60^, ^63^ The pharmacokinetic parameters wash in constant *K*_trans_ and distribution volume of contrast agent *V*_e_ and the model‐free parameters initial slope of enhancement, area under the curve, and maximum enhancement/peak concentration have been found to be elevated in inflamed versus normal bowel segments.^22^, ^26^, ^41^ Lee et al^24^ compared active and inactive CD groups and found that *K* ^trans^ was significantly higher in the active compared to the inactive group. There was also correlation between *K* ^trans^ and clinical score, endoscopic scores, and CDMI.

In CD, both inflammation and fibrosis demonstrate different enhancement pattern on contrast‐enhanced MRE; overall the fibrotic lesions enhance less (histological changes in fibrosis are hypothesized to retard blood flow).^64^ Tielbeek et al^42^ demonstrated positive correlation between the model‐free parameters maximum enhancement and initial slope of enhancement with histopathological grading of inflammation in surgical specimens. This study found that the same two parameters were elevated in fibrostenosis, highlighting the difficulty with separating inflammation and fibrosis when they coexist in the same segment and suggesting that DCE‐MRI parameters may have limited utility in distinguishing between them.

Thus far, DCE‐MRI in CD has demonstrated mixed results in its use as a biomarker of fibrosis. Rimola et al^23^ found that the degree of fibrosis in surgical specimens correlated with the pattern of enhancement at 7 minutes after contrast administration and the model‐free percentage of enhancement gain between 70 seconds and 7 minutes. Percentage of enhancement gain could distinguish between mild–moderate and severe fibrosis deposition with a sensitivity of 0.94 and a specificity of 0.89 in small bowel disease. However, a recent multicenter study of 60 patients with CD using histological samples as the reference standard found only weak association between enhancement gain and bowel wall fibrosis and no association with inflammation.^25^ Studies comparing DCE‐MRI with other techniques such as IVIM‐DWI^34^ and T2*^35^ have found that the latter techniques may be superior for determining the severity of fibrosis, with the added benefit that they do not require administration of intravenous contrast. A further potential limitation of DCE‐MRI in clinical practice is the requirement for technical consistency, particularly in acquisition timings following the bolus of contrast.

### Magnetization Transfer Ratio

Magnetization transfer imaging (MTI) is an MRI technique that generates image contrast based on interactions between the protons of free water and those immobilized in large macromolecules, such as collagen. In MTI, two gradient‐echo datasets are acquired, with and without application of an off‐resonance RF pulse. The off‐resonance pulse saturates the protons in macromolecules (but not those in free water). The saturated macromolecule‐bound protons partially transfer their magnetization to protons in the free water, so some free water protons become saturated. When the second (excitation) RF pulse is applied, the signal from the free water is reduced due to the pre‐saturation of some free‐water protons. The difference between the signals achieved with and without the off‐resonance pulse can be compared and is the so‐called the magnetization transfer ratio (MTR). The magnetization transfer effect varies in different tissues (depending on the degree of interaction between the macromolecule and free water pools) and increases with the number of protons in macromolecules that transfer some of their magnetization to protons in the free water. The effect size (the MTR) represents a measure of the proportion of macromolecules, for example collagen, in the physiological environment.

Bowel wall fibrosis in CD is characterized by excessive collagen accumulation in the extracellular matrix^4^ and the concentration of collagen in the fibrotic intestinal walls therefore determines the MTR, with a greater MTR with increasing bowel fibrosis. Several relatively small studies using histopathology as the gold standard have demonstrated the feasibility of MTR for identifying intestinal fibrosis and shown correlation between the MTR and histologic bowel fibrosis scores.^36^, ^37^ Li et al^32^ also found that MTR outperformed ADC and DCE in detecting and distinguishing different degrees of bowel fibrosis. However, in a multicenter study of 60 patients, Coimbra et al^25^ found weak association between MTR and fibrosis, raising questions about its potential utility.

A limitation of MT imaging is that physiological macromolecules within the normal bowel wall, such as smooth muscle, would also increase the MTR and so influence the accurate assessment of the severity of bowel fibrosis. The potential clinical utility of MT imaging is limited by is low signal‐to‐noise ratio and high specific absorption rate. A further limitation of MTR is variability in reported interobserver agreement (see Table 4).

### Motility

**Figure 5:**
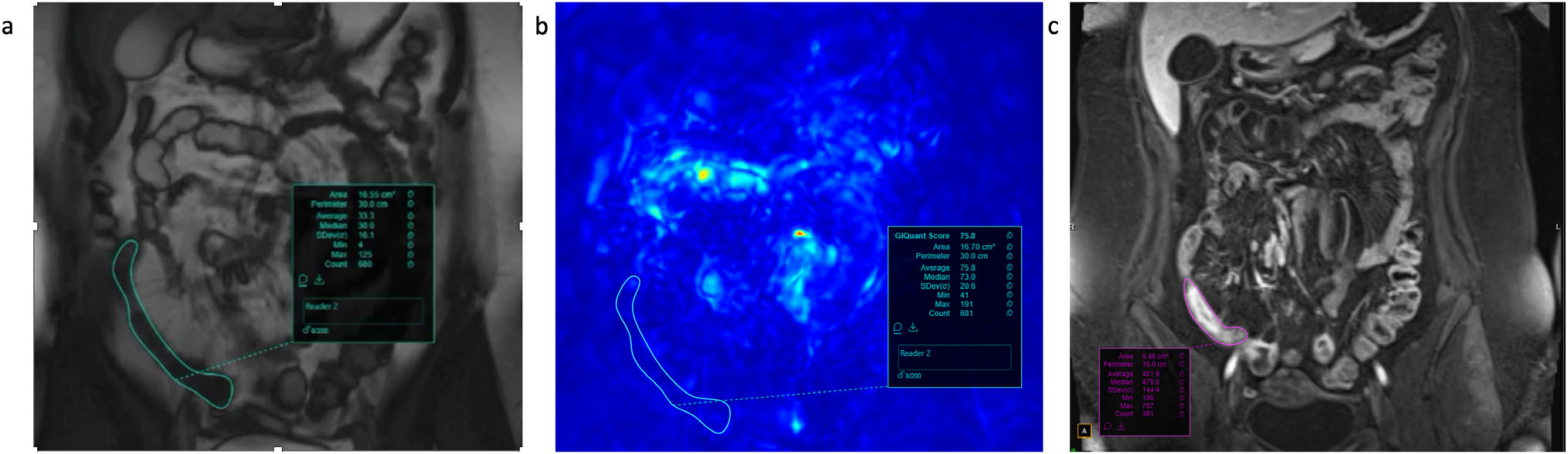
Motility MRI in a patient with active terminal ileitis due to CD. (**a**) Reference balanced steady‐state free precession anatomical image with ROI in the area of involvement in the TI; (**b**) motility MRI parametric map. Red depicts areas of high motility while blue areas of low motility. The motility map indicates reduced motility in the affected region. The GIQuant (intestinal motility) score was 75.8; (**c**) T1 fat‐saturated postcontrast image for reference, showing enhancement of the inflamed TI. CD = Crohn's disease; ROI = region of interest; TI = terminal ileum.

An alternative to measuring disease activity by evaluating bowel structure is to evaluate bowel function in the form of segmental motility. Bowel motility is a complex neuromuscular function, but in brief, normal bowel demonstrates smooth peristaltic waves whereas in diseased bowel such as in CD, inflammation, and fibrosis lead to reduction in and disorder of peristalsis.^73^ Bowel motility can be assessed subjectively using ultrasound but advances in acquisition and postprocessing now enable rapid MRI‐based quantification of motility with minimal user input. MR protocols are based on fast T2‐weighted SSFP or echo‐planar sequences with 1 image frame/second for at least 15–20 seconds^74^ (Fig. 5).

Terminal ileal motility scores are negatively correlated with histological disease activity and show excellent agreement with the MaRIA score.^33^ Improvements in motility reflect treatment response in CD with motility post‐therapy shown to be 93% sensitive and 77% specific for clinical response to anti‐TNFα agents and motility may be more sensitive to early treatment response than morphological assessment.^67^ Failure to respond (i.e., a decrease or no change in motility) is potentially a hallmark of fibrosis predominant or treatment resistant disease. Of note, current studies have focused on inflammation in CD and to date, there have been no studies assessing the influence of fibrosis on motility. A potential disadvantage of this technique is that analysis requires specialist postprocessing software.

### Elastography

Magnetic resonance elastography can be used to assess tissue stiffness and is validated for quantification of hepatic fibrosis.^75^ MR elastography uses propagating shear waves that induce micromovements inside the tissue of interest. These shear waves are depicted with a phase‐contrast sequence and through analysis of wave images indicating the propagation of shear waves in the tissues, a stiffness map can be generated.

A few studies have investigated the use of MR elastography for the detection of intestinal fibrosis in patients with CD. In a study of 69 patients with CD, Avila et al^39^ found that the stiffness value measured by MR elastography correlated with the degree of fibrosis (as measured by visual analog score by a radiologist) and that greater bowel stiffness was associated with an increased risk of adverse clinical events such as abdominal surgery, hospitalization, or consultation at emergency department for abdominal pain or digestive occlusion. A further study of multifrequency MR elastography found that shear‐wave speed and loss angle (representing stiffness and sloid‐fluid behavior, respectively) could predict the presence of histologically diagnosed inflammatory bowel disease.^40^

While MR elastography demonstrates some potential for assessment of intestinal fibrosis, there have only been a few small studies and this technique requires further validation.

A further consideration is the requirement for specialist equipment to acquire the MR elastography images.

### Combining qMRI Parameters to Improve Performance

As discussed earlier, qMRI is intrinsically designed to separate out the information acquired by MRI so that different parameters give information on individual processes. For example, the potential confounding effect of fat on T2 measurements can be accounted for by measuring T2 water and fat fraction independently with the aim of giving a more accurate characterization of edema. However, combining QIBs which may be derived from a single technique or from different qMRI techniques in combination could potentially enhance performance further. Hectors et al^26^ investigated the combined use of IVIM and DCE‐MRI in differentiating normal and inflamed bowel segments (defined as bowel thickness >3 mm on T1‐weighed images) in patients with CD. Both pharmacokinetic (*K* ^trans^ [AUC = 0.694] and *v*_e_ [AUC = 0.704]) and model‐free (peak concentration [AUC = 0.733], upslope [AUC = 0.693], area under the curve [AUC = 0.733]) were significantly increased and the IVIM parameters perfusion fraction (AUC = 0.734) and ADC (AUC = 0.800) were significantly decreased in abnormal bowel segments. Combining multiple parameters (*K* ^trans^ + *v*_e_+$\;f_{\text{IVIM}}$ + ADC) showed highest AUC (0.963), demonstrating the potential of multiparametric MRI in assessing disease activity in CD. Mao et al^76^ found that a combination of ADC_fast_, *K* ^trans^, and *K*_ep_ gave the highest AUC (0.974) for distinguishing between active and inactive disease and outperformed the MaRIA score (AUC = 0.902). Oto et al^22^ found that combining *K* ^trans^ and ADC data gave an AUC of 0.95 for detection of active inflammation (defined as the presence of mural hyperenhancement); for reference the AUC for the individual parameters *K* ^trans^ and ADC were 0.92 and 0.92, respectively.

A limitation of combining multiple parameters is that this will always improve performance on the cohort used to develop the model, but the model is not generalizable to other cohorts (this can be seen as overfitting to the “training” dataset). Further studies are therefore necessary to validate these findings in additional cohorts.

## Considerations for Clinical Translation

The qMRI techniques described earlier show potential in the diagnosis and monitoring of CD in the small bowel and are at different stages of development. For example, DWI is acquired as part of clinical sequences in CD but the quantitative information from the ADC is not routinely interrogated, whereas other methods are currently only acquired as part of research studies. The process of developing a qMRI technique and the relevant imaging biomarker takes time as it involves many steps^77^ and there are additional practical considerations when imaging patients. Some of these are considered in the following sections.

### A Robust Clinical Reference Standard

The Quantitative Imaging Biomarker Alliance (QIBA) has published guidelines for assessment of the technical performance of QIBs in terms of their repeatability, reproducibility, and accuracy^78^ (repeatability and reproducibility are discussed in the next section). Accuracy refers to the degree of bias and linearity of the measured QIB relative to the true or accepted reference measurements.

The reference standard for quantifying fibrosis in CD is whole specimen histopathological samples. While these can be obtained from patients undergoing bowel resections, there are a number of practical limitations to this: acquiring MRI scans just before surgery may be difficult, matching of the histological specimen to the imaging can be challenging, and as bowel resection is often only udertaken for refractory disease, it is not possible to use this for disease monitoring. Once histopathological samples are acquired, inflammation and fibrosis can be assessed using various scoring systems, for example, the acute inflammatory score^79^ and the Chiorean three‐point scale for fibrosis grading.^80^

Limitations of the histopathological scoring systems include variation in the scoring system used between studies leading to difficulties in reliable comparisons; lack of fully validated scoring systems particularly in fibrosis; and heterogeneity of the type of pathological process with inflammation and muscular hypertrophy acting as confounding factors when assessing fibrosis. Another option for histopathological sampling is at endoscopy. However, the invasive nature of endoscopy precludes repeated assessment and is unable to access much of the small bowel. A further limitation of histopathological samples acquired at endoscopy is that they may not demonstrate the full thickness of the involved wall and there is the possibility of missing involved areas as only small samples are taken.

Where histopathological samples are not available, surrogate reference standards may be used, for example, the CDEIS score,^13^, ^14^ clinical grading of images by experienced readers,^38^ the CDAI,^81^ or validated MRE activity scores. MRE scoring systems have demonstrated good correlation with histopathological samples and have been found to be highly sensitive for detecting active disease in the terminal ileum but generally have low specificity.^12^ Some of the low specificity is purported to result from endoscopic skipping (mentioned earlier) where some of the active inflammation is not visible at endoscopy; in this circumstance, MRE would be more sensitive than endoscopy. Clinical assessment and diagnosis or comparison with signal abnormalities on conventional MR may therefore be used as surrogate reference standards where they offer a method to ensure that the expected effect (eg, more active disease score) is present, noting that these are imperfect methods which may be less sensitive to the subtle inflammation which qMRI aims to detect.

### Repeatability and Reproducibility of qMRI Methods

The repeatability of qMRI techniques is a measurement of variability in data acquired from a series of measurements under identical conditions (for example scanning the same patient twice on the same scanner). The repeatability of measurements of T1 and T2 relaxometry, IVIM‐DWI, and MT in the small bowel has been assessed in 10 healthy volunteers; results were variable for the different parameters but demonstrated excellent repeatability for ADC, MTR, and T1.^68^ Small bowel motility measurements have also demonstrated varying repeatability in volunteers with one study demonstrating a coefficient of variation of 4.9%^82^ and another a coefficient of variation of 34.6% in subjects prepared with mannitol and 23.7% in unprepared subjects.^83^ A summary of qMRI repeatability measurements in the small bowel is provided in Table 3.

**Table 3:** Summary of Repeatability Measurements for qMRI Techniques in Crohn's Disease

Study	Technique	Parameter	Repeatability
Menys et al^82^	Motility	Motility index	CoV 4.9% Bland–Altman mean difference − 0.0025, 95% limit of agreement ±0.044
Alyami et al^68^	Relaxometry	T1	CoV 8%
		T2	CoV 21%
	IVIM‐DWI	ADC	CoV 5%
		D	CoV 10%
		PF	CoV 20%
		D*	CoV 31%
	Magnetization transfer imaging	MTR	CoV 7%
De Jonge et al^83^	Motility	Motility index	CoV 34.6% (mannitol prepared subjects) CoV 23.7% (unprepared subjects)
Reiter et al^40^	Elastography	SWS	Standard deviation 1.05 ± 0.03 m/second
		Loss angle	Standard deviation 0.57 ± 0.03 rad

The repeatability (test–retest) of qMRI techniques refers to variability in data acquired from a series of measurements under identical conditions (for example scanning the same patient twice on the same scanner).

ADC = apparent diffusion coefficient; CoV = coefficient of variation; D = diffusion coefficient; D* = perfusion coefficient; DWI = diffusion‐weighted imaging; IVIM = intravoxel incoherent motion; MTR = magnetization transfer ratio; PF = perfusion fraction; SWS = shear‐wave speed.

Reproducibility refers to variability in measurements made on the same subject but under different conditions, for example, different scanner, location, field strength, or postprocessing software. Commonly, the initial technical validation is achieved by using a test object (phantom) which can be imaged on different scanners at different sites from different vendors and with different field strengths. Any bias in the measurements can be identified and imaging protocols can be adjusted accordingly to improve reproducibility. Phantom studies have demonstrated coefficients of variation of 8.21% (at 1.5 T) and 5.46% (at 3 T) for T1 quantification used in DCE‐MRI protocols,^84^ <4.0% for T1 quantification after B1 correction,^85^ and <7.3% for R2*.^85^ In two phantom studies of the reproducibility of ADC, the coefficients of variation were <3.98%^85^ and <3%.^86^

Further studies will be required to demonstrate the repeatability of candidate qMRI measures in CD.

### Intra‐ and Interobserver Variability of MRI Measurements

Intra‐ and interobserver variability of qMRI measurements refer to differences in measurements by the same observer on different occasions and differences between multiple observers, respectively. Good agreement between observers is vital when considering the clinical translation of qMRI techniques. Agreement data from studies are summarized in Table 4. It should be noted that a number of studies made measurements in consensus so did not assess observer agreement.

**Table 4:** Summary of Agreement Statistics From qMRI Studies in Crohn's Disease

Study	qMRI Technique	Parameter	Intraobserver Agreement	Interobserver Agreement
Li et al^27^	DWI	ADC	ICC 0.831	
Watson et al^65^	DWI	ADC	Lin CC 0.844	ICC 0.51
Huh et al^66^	DWI	ADC		ICC 0.918
Buisson et al^15^	DWI	ADC		Lin CC 0.71
Li et al^30^	DWI	ADC		ICC 0.97
Caruso et al^28^	DWI	ADC		*κ* = 0.861
Plumb et al^67^	Motility^67^	Motility index		ICC at baseline 0.65 ICC post‐treatment 0.71
Zhang et al^34^	IVIM‐DWI	*f*		ICC 0.851
		D		ICC 0.855
		D*		ICC 0.719
		ADC		ICC 0.832
Huang et al^35^	Relaxometry	T2*		ICC 0.893
Lee et al^24^	DCE‐MRI	*K* ^trans^		ICC 0.912
		*K* ^ep^		ICC 0.876
		*v*_e_		ICC 0.861
Fang et al^37^	Magnetization transfer imaging	MTR		ICC 0.899
Fang et al^37^	% Mucosal enhancement gain	30 seconds vs. 7 minutes		ICC 0.818
		70 seconds vs. 7 minutes		ICC 0.629
Alyami et al^68^	Relaxometry	T1	ICC 0.33	ICC 0.55
		T2	ICC 0.91	ICC 0.89
	IVIM‐DWI	ADC	ICC 0.85	ICC 0.76
		D	ICC 0.83	ICC 0.86
		*f*	ICC 0.05	ICC 0.41
		D*	ICC 0.22	ICC 0.14
	Magnetization transfer imaging	MTR	ICC 0.32	ICC 0.08
Choi et al^69^	Motility	Motility index		ICC 0.987
Avila et al^39^	Elastography	Stiffness		ICC 0.95
Reiter et al^40^	Elastography	Shear wave speed^a^		ICC 0.78
		Loss angle^b^		ICC 0.61
Study	Conventional MRI feature on MRE	Overall assessment on MRE with conventional sequences		Inter‐observer agreement
Tielbeek et al^70^	Wall thickness			ICC 0.69
	Presence of edema			*κ* = 0.66
	Enhancement pattern			*κ* = 0.62
	Length of disease segment			*κ* = 0.62
Bhatnagar et al^10^		Presence of disease		New diagnosis: 68% (*κ* = 0.36) Relapsed disease: 78% (*κ* = 0.56)
		Disease extent		New diagnosis: 43% (*κ* = 0.14) Relapsed disease: 53% (*κ* = 0.07)
Church et al^71^	Reduction in motility			*κ* = 0.69
	Wall DWI hyperintensity			*κ* = 0.64
	Wall T2 hyperintensity			*κ* = 0.55
	Wall enhancement			*κ* = 0.44
Jensen et al^72^	Bowel wall thickening			*κ* = 0.43
	Bowel wall hyperenhancement			*κ* = 0.50
		Presence of disease		*κ* = 0.48

Intra‐ and interobserver agreement of qMRI measurements refers to differences in measurements made by the same observer on different occasions and between different observers, respectively. Good agreement is vital when considering the clinical translation of qMRI techniques. For reference inter‐observer agreement statistics for conventional features/overall impression on MRE are shown in the second part of the table.

ADC = apparent diffusion coefficient; *D* = diffusion coefficient; D* = perfusion coefficient; DCE‐MRI = dynamic contrast enhanced MRI; *f* = perfusion fraction; DWI = diffusion‐weighted imaging; ICC = intraclass correlation coefficient; IVIM = intravoxel incoherent motion; *K* ^ep^ = wash‐out constant; *K* ^trans^ = volume transfer constant between the intravascular space and extravascular space; Lin CC = Lin concordance coefficient; MTR = magnetization transfer ratio; *v*_e_ = volume of extravascular space per unit volume of tissue.

^a^ Represents stiffness.

^b^ Represents solid–fluid behavior.

The reported interobserver agreement for many of the qMRI biomarkers varies among studies. For example, interobserver agreement of ADC measurements has been reported as moderate in one study (ICC 0.51)^65^ and excellent in other studies (ICC 0.918,^66^ 0.97^32^). Observer agreement also varies for each of the IVIM parameters. Across two studies, the highest interobserver agreement was seen for the diffusion coefficient (ICC 0.855^34^ and 0.86^68^) and the ADC (0.832^34^ and 0.76^68^). In a single study, intraobserver agreement was excellent for ADC and the diffusion coefficient (ICC 0.85 and 0.83, respectively)^68^ and poor for the other IVIM parameters. For motility measurements the ICC ranges from 0.65^67^ to 0.987.^69^

These differences may reflect differences in the experience level of observers or in the subject cohorts (eg, some studies have assessed intra and interobserver variability in healthy subjects whereas others have used patients with CD). The majority of studies use ROIs placed on the area of the bowel of interest to extract qMRI measurements. Typically, studies of qMRI in CD use manual ROIs and therefore rely on observer expertise and mean that there is the potential for subjectivity. Interobserver variability of the measurements arises when ROIs are placed in different locations; this could be because of failure to identify an anatomical landmark (such as the terminal ileum) accurately, inhomogeneous inflammation with areas of intervening normal bowel meaning that different inflamed sites are sampled, or challenges in accurate placement of the ROI caused by bowel motility.

Advances in segmentation techniques may reduce variability and the amount of time taken for image analysis. For example, postprocessing of the MR images incorporating motion correction reduces distortions caused by breathing and peristalsis.^53^ Semi‐ or complete automation of bowel wall identification and subsequent measurement extraction from parametric maps has the potential to reduce observer variability and accelerate image analysis. For example, semi‐automatic bowel wall identification and measurement extraction from T2 maps of the small bowel has demonstrated good inter‐ and intraobserver agreement.^68^ Notably, agreement was poor for MTR which required complete manual ROI definition.

### Practical Considerations When Imaging Patients

Ultimately the aim of developing qMRI techniques is that they may be used in routine clinical care for patients. This means that the technique must be acceptable to patients, for example it must not considerably increase the duration of the MRI scan and should not involve long periods of breath holding. Minimizing the duration of the scan also means that images are less likely to be affected by motion, increasing the accuracy of the quantitative measurements. DWI and MR relaxometry‐based techniques do not require the administration of intravenous contrast which is a further advantage when designing imaging protocols used on young patients who will have many repeated examinations over their lifetime. This also removes the risk of allergic reactions to contrast media and has the potential to reduce costs. A final consideration for translation to clinical use is the availability of the quantitative imaging techniques; the data required for most of the candidate techniques can be acquired relatively easily on modern MRI scanners, but the postprocessing will need to be integrated into the scanner systems to enable routine clinical use.

## Future Directions

Ultimately, qMRI and imaging biomarkers offer promise both to inform clinical decision making and act as robust endpoints in clinical trials investigating new treatments, but further development is required to materialize these goals.

Manual segmentation of areas of involved bowel is fairly time consuming and measurements differ between observers. Advances in deep learning and artificial intelligence techniques enabling automated image registration and measurement extraction could result in efficient and consistent image analysis that also minimizes the interobserver variability. Recent studies have demonstrated the feasibility of segmentation of the bowel wall^87^ and segmentation of affected regions of bowel^88^ on MRI in CD using deep learning. These techniques could be incorporated into clinical workflows evaluating CD.

## Summary

Quantitative MRI offers potential for the accurate assessment of the small bowel in CD with separate measurements of inflammation and fibrosis. This could enable early identification of disease relapse and early and precise of treatment with the aim of limiting long‐term damage and inducing sustained remission. However, as yet, none of the candidate qMRI techniques have reached widespread clinical use. The choice of a qMRI technique and biomarker for further development will be influenced by availability, preliminary results, and perceived translational potential. MR relaxometry‐based techniques which are widely available, have high signal‐to‐noise ratios, and do not use intravenous contrast may be a pertinent choice for further investigation in this context.
